# Neurodevelopmental Implications Underpinning Hereditary Spastic Paraplegia

**DOI:** 10.1111/cns.70260

**Published:** 2025-02-11

**Authors:** Yiqiang Zhi, Yan Shi, Danping Lu, Dan Xu

**Affiliations:** ^1^ Fujian Key Laboratory of Molecular Neurology, Institute of Neuroscience, School of Basic Medical Sciences Fujian Medical University Fuzhou China; ^2^ Fujian Key Laboratory of Molecular Neurology, Institute of Neuroscience Fujian Medical University Fuzhou China; ^3^ College of Life Sciences Fujian Agriculture and Forestry University Fuzhou China

**Keywords:** clinical features, expression trajectories, hereditary spastic paraplegia, neurodegenerative disorders, neurodevelopmental mechanism, protein function

## Abstract

**Background:**

Hereditary spastic paraplegia (HSP) is a group of rare genetic neurodegenerative disorders characterized by corticospinal tract abnormalities. But frequently, abnormalities of proteins implicated in HSP have been identified in brain disorders of childhood, raising the possibility that early brain developmental mechanism underlying HSP.

**Results and Conclusions:**

Here we summarized the clinical features of 89 HSP subtypes and found most have onset of symptoms earliest reported in infancy or early childhood. Importantly, HSP patients showed early brain developmental related phenotypes such as microcephaly, ventricular enlargement, and corpus callosum dysplasia. In addition, the expression trajectories analysis showed HSP genes were diffusely expressed across all human prenatal cortical regions and most genes enriched from post‐conception weeks 8–24, periods characterized by neuro progenitor proliferation and neurogenesis. Furthermore, studies utilizing patient derived induced pluripotent stem cells (iPSCs)/organoids and mouse models have suggested that most HSP proteins play either direct or indirect roles in the development of the central nervous system. Therefore, HSP possesses a neurodevelopmental aspect and is not merely a degenerative disease, which may aid in better understanding the pathogenesis of this disease.

## Graphical abstract

Hereditary spastic paraplegia (HSP) is a group of rare genetic neurodegenerative disorders. This review comprehensively outlines the clinical manifestations, gene expression trajectories, and protein function of HSP subtypes from the perspective of neurodevelopment. Insights into the early developmental mechanism underlying HSP may ultimately enhance the understanding of this disease and lead to the discovery of novel treatments.

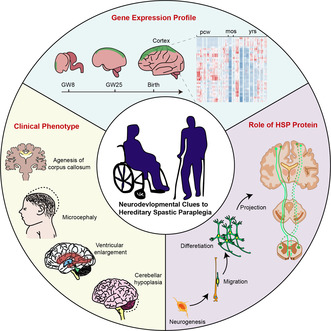

## 1 Introduction

Converging evidence suggests that brain structure alterations may proceed overt cognitive impairment or motor disability in neurodegenerative diseases by several decades. Magnetic resonance imaging measurements of white matter myelin water fraction and gray matter volume in healthy infant carriers and noncarriers of the apolipoprotein E (APOE) ε4 allele demonstrates some of the earliest brain changes associated with the genetic predisposition to Alzheimer's disease (AD) [1, 2]. The measures of growth are abnormal in child and adolescent carriers of mHTT, decades before Huntington's disease (HD) onset [3]. Neocortical sulcal measures indicate coexistence of abnormal neurodevelopmental and neurodegenerative processes in HD [4]. Lower intracranial volume in subjects with prodromal HD compared with controls lends support to the theory that abnormal brain development may be a precursor to neurodegeneration in HD [5]. Thus, Mehler and Gokhan propose that neurodegenerative diseases originate from abnormal brain development and are atypical neurodevelopmental disorders [6].

Recently, increasing number of studies has begun to focus on exploring the developmental mechanisms in neurodegenerative diseases. Although AD is considered to be an aging‐related neurodegenerative disorder, the most common used animal model 5XFAD mice express mutant forms of APP/PS1 throughout the life span. Disrupted maturation of prefrontal Layer 5 neuronal circuits were detected in 5XFAD mice [7], indicating early developmental defects of cortical circuits may contribute to the age‐dependent synaptic pathology and neurodegeneration later in life. In a study of spinal cerebellar ataxia, overexpression of mutant ATXN1 protein in a mouse model stimulated the proliferation of stem cells in the developing cerebellum and excessive differentiation into GABAergic inhibitory neurons, leading to functional disruption of Purkinje cells and causing cerebellar impairment resulting in ataxia [8]. In one research on Parkinson's syndrome, homozygous mutations in DNAJC6 could lead to widespread neurodevelopmental defects, including impairments in dopaminergic neurons and functional disturbances in ventral midbrain‐like organ structures, as observed in the creation of induced iPSCs and brain‐like organ models [9]. Research utilizing mouse embryonic models and human fetal tissues in studies of HD has shown that HD impacts neurodevelopment, specifically inducing defects in neuroepithelial cell junctions, disrupting interkinetic nuclear migration (INM), and altering the cell cycle [10]. In addition, HTT protein is required for multipolar bipolar conversion and projection neuron migration, loss of HTT during embryonic period affects morphology of adult neurons [11]. Nonetheless, mouse studies show that either expressing mHTT or depleting wild‐type (WT) HTT for only 2–3 weeks after birth is sufficient for the mice to later develop the hallmark features of HD pathology [12]. Therapeutic effects on sensory, motor, and cognitive functions can be observed and preserved in HD adult mice when GABA receptor agonist pharmacological interventions are administered to HD neonatal mice [13]. More importantly, selective expression of mutant huntingtin during development recapitulates characteristic features of Huntington's disease. Taken together, numerous proteins, which are hypothesized to be crucial in the progression of neurodegeneration, exert either direct or indirect influences on the development of the central nervous system.

Hereditary spastic paraplegia (HSP), also known as familial spastic paraparesis (SPG), refers to a group of inherited disorders that characterized by spastic paraplegia of the lower limbs [14]. Some patients may also experience other neurological symptoms, such as ataxia, muscle spasm, urinary incontinence, and intellectual impairment. Clinically, HSP/SPG can be divided into pure and complex types. Pure HSP mainly characterized by slowly progressive spastic weakness of the lower limbs and spasticity. Complicated HSP is associated with additional neurologic symptoms, including mental retardation, epilepsy, multiple psychiatric disorders, retinal dysfunction, etc. In most cases, pure HSP shows autosomal dominant (AD) or X‐linked and complicated HSP shows autosomal recessive (AR) inheritance [15]. Currently, only a limited number of HSP types, such as SPG3A/ATL1, SPG7/PGN, SPG9/ALDH18A1, SPG18/ERLIN2, SPG30/KIF1A, SPG58/KIF1C, and SPG72/REEP2, have been demonstrated to exhibit both AD and AR patterns of inheritance. Interestingly, a recent study revealed that the p.V168M mutation in *ERLIN2* leads to a phenoconversion, manifesting as amyotrophic lateral sclerosis (ALS) in the second generation, pure HSP in the third generation, and complicated form of HSP in the fourth generation [16]. Therefore, there exists a complex mechanism underlying the multifaceted nature of certain types of HSPs.

HSP exhibits a high degree of heterogeneity, encompassing a diverse array of genetic subtypes, and is characterized by substantial variations in both the age of onset and clinical manifestations [17]. Although HSP was considered as a neurodegenerative disorder, it can present in neonatal, infancy, childhood, adolescence, or adulthood. Thus, it is crucial to understand the early developmental mechanisms associated with HSP, which will facility to identify the best targets and approaches toward this disease. Here we summarized the clinical phenotypes, expression trajectory, and protein functions of HSP from the perspective of neurodevelopment. We believe that the studies of neurodevelopmental implications of HSP will provide a new perspective on the pathogenesis of this disease.

## 2 The Clinical Features and Neocortical Morphometry Highlights the Presence of Brain Developmental Phenotypes in HSP

**FIGURE 1:**
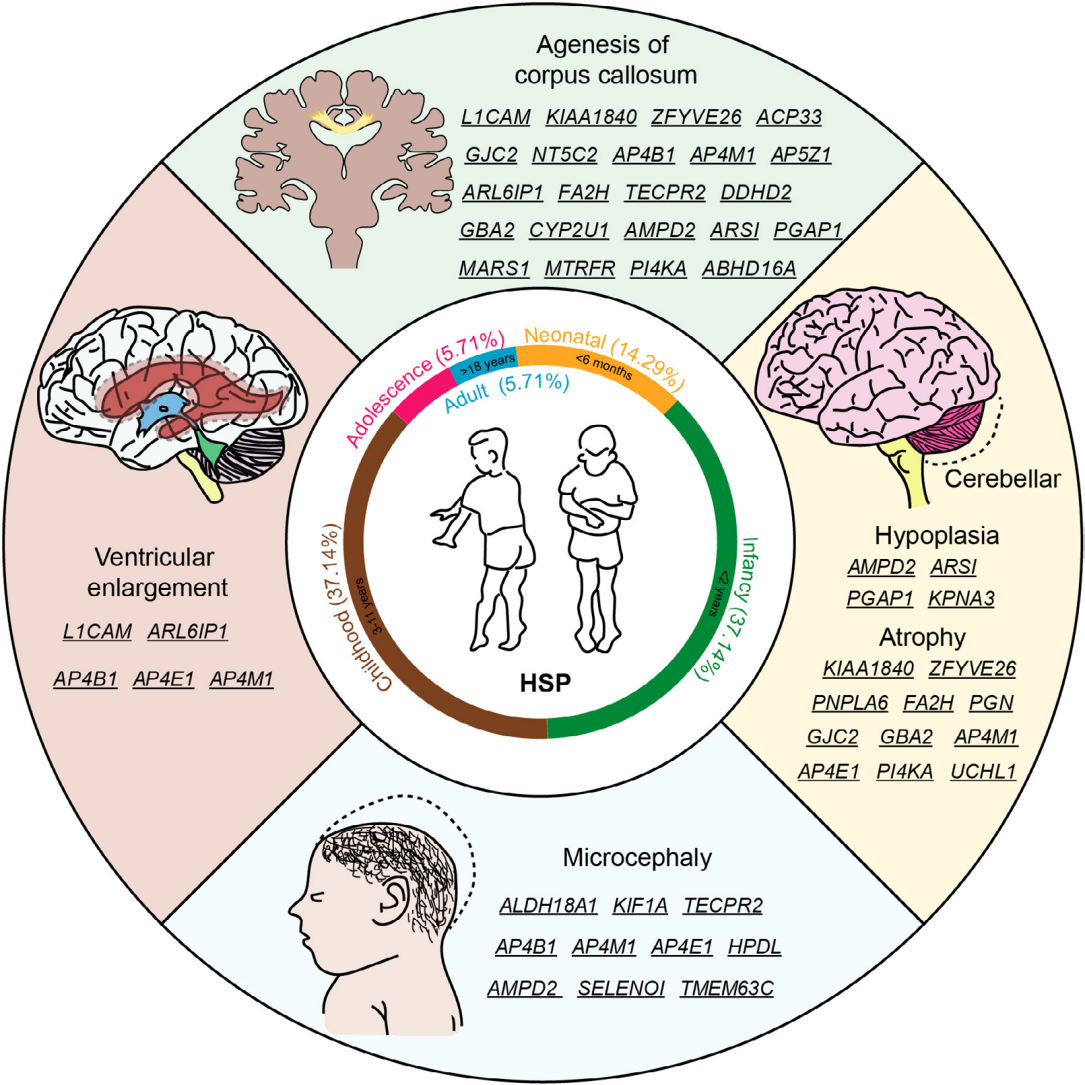
HSP patients showed symptoms of early‐onset and neurodevelopmental aspects. HSP patients exhibit their earliest symptoms at various stages, including neonatal, infancy, childhood, adolescence, and adulthood. Furthermore, dysfunction of certain HSP proteins can lead to developmental disorders, such as microcephaly, corpus callosum agenesis defect, ventricular enlargement, and cerebellar hypoplasia.

The genetic background of HSP is complex, which related to the type and location of gene mutations. According to the Online Human Mendelian Inheritance Database (OMIM), 89 spastic paraplegia gene (SPG) subtypes and 76 causing genes of HSP have been identified (Tables S1 and S2). We analyzed the clinical phenotypes of the 89 identified subtypes and discovered that 14.29% subtype of HSP patients with symptoms earliest reported on neonatal (from birth to up to 6 months), 37.14% subtype of HSP with symptoms earliest reported on infancy (birth to 2 years old), or on childhood (3–11 years old), and only 5.71% subtype of HSP with symptoms earliest reported on adolescents (12–18 years old) or adult (older than 18 years old) [18]. Moreover, until now over 35% HSP subtypes were reported exhibiting developmental abnormalities, such as microcephaly, corpus callosum agenesis defect, ventricular enlargement, and cerebellar hypoplasia (Table S1 and Figure 1).

### 2.1 Microcephaly

Primary Microcephaly is a neurodevelopmental disorder defined as a significant reduction (more than two standard deviations below the mean) in the occipitofrontal head circumference compared with controls matched for age and sex [19]. Microcephaly can be developmental resulting from abnormalities of neural progenitor proliferation, differentiation and cell death during brain development [20]. So far, mutations in 10 genes associated with HSP have been identified as also causes of microcephaly.

Mutations in the aldehyde dehydrogenase 18 family member A1 (*ALDH18A1*) gene result in delta‐1‐pyrroline‐5‐carboxylate synthetase (P5CS) deficiency, were known to cause a condition presenting some phenotypic resemblance with SPG9, including a neurodevelopmental syndrome with microcephaly [21]. Kinesin family member 1 (KIF1A /SPG30) is a neuron‐specific member of the kinesin‐3 family of ATP‐dependent microtubule molecular motor proteins. KIF1A‐associated neurological disorder has a broad phenotypic spectrum, which include spasticity, neurodevelopmental delay, intellectual disability, autism spectrum disorder, microcephaly, progressive spastic paraplegia, autonomic and peripheral neuropathy, optic nerve atrophy, cerebral and cerebellar atrophy, and seizures [22]. Interestingly, mutations in genes encoding members of the adaptor‐related protein complex 4 (AP4B1/SPG47, AP4M1/SPG50, and AP4E1/SPG51) have been associated with microcephaly and severe cognitive impairment in agreement with their role in vesicular trafficking during the development of the central nervous system [23, 24, 25, 26, 27]. Tectonin beta‐propeller repeat containing 2 (TECPR2/SPG49) was identified as an interactor of the ATG8 family proteins, which play key roles in autophagy. *TECPR2‐*related hereditary sensory and autonomic neuropathy with intellectual disability is characterized by developmental delay, microcephaly, and subsequent intellectual disability, behavioral abnormalities [28]. Biallelic variants in adenosine monophosphate deaminase 2 (*AMPD2*/*SPG63*) caused pontocerebellar hypoplasia with unique combination of postnatal microcephaly, hypoplastic cerebellum and pons, and hypoplastic or absent corpus callosum [29]. Selenoprotein I (SELENOI) is involved in phospholipid biosynthesis and neurodevelopment. Mutation in the SELENOI caused a complicated form of HSP (SPG81), associated with variable features including microcephaly [30]. 4‐Hydroxyphenylpyruvate dioxygenase like (*HPDL/SPG83*) biallelic mutation leads to a range of neurological phenotypes, which included spastic tetraplegia, microcephaly, brain atrophy, epilepsy, and severe intellectual and motor disability [31]. Biallelic variants in the transmembrane protein 63C (*TMEM63C*) gene, encoding a predicted osmo sensitive calcium‐permeable cation channel, cause individuals with SPG87 associated with microcephaly in some, but not all cases [32].

### 2.2 Agenesis of Corpus Callosum

The corpus callosum is the primary commissural region of the brain consisting of white matter tracts that connect the left and right cerebral hemispheres [33]. The interhemispheric coordination of motor, sensory, and cognitive functions is realized by information exchange through ~200 million axons in the main tract [34]. Corpus callosum development starts from the 8th week of fetal life and continues throughout the gestation period. Agenesis/hypoplasia (imperfect development) of the corpus callosum is one of the most common abnormalities of brain development. 22.45% of HSP subtypes showed corpus callosum abnormalities including thin corpus callosum and corpus callosum hypoplasia, while it remains unclear whether the thin corpus callosum phenotype is a result of congenital thinning/hypoplasia or whether it is related to progressive atrophy.

Mutations in the L1 cell adhesion molecule (L1CAM/SPG1), an axonal glycoprotein involved in neuronal migration and differentiation, have been identified in the following various X‐linked neurological disorders: congenital hydrocephalus; mental retardation, aphasia, shuffling gait, adducted thumbs syndrome, and agenesis of the corpus callosum [35, 36]. SPG11, which is caused by *KIAA1840* gene (enocde spatacsin) mutations, is the most common subtype of AR HSP, accounting for ~20% of patients and up to 60% of patients with thin corpse callosum [37]. SPG15, the second most abundant AR case, encodes the ZFYVE26 protein and is involved in autophagy lysosome formation and axon growth of spinal motor neurons. Neuroimaging showed thin corpus callosum and generalized cortical atrophy [38]. Fatty acid 2‐hydroxylas (*FA2H*/*SPG35*) is a gene linked to fatty acid hydroxylase. SPG35 patients usually develop in childhood with spastic paralysis and cerebellar ataxia phenotypes, and imaging showing complex changes including corpus callosum thinning, cerebral white matter lesions and pontocerebellar atrophy [36, 39].

HSP patients with problems in the development of the corpus callosum are often accompanied by other neurodevelopmental manifestations as well, such as enlarged ventricles and neural atrophy, which can lead to mental retardation, cognitive impairment, and other problems. By categorizing and analyzing these underlying causes and delving into their deeper molecular mechanisms, we may uncover potential links between neurodevelopment and neurodegenerative diseases and also help to provide precise treatment for patients.

### 2.3 Cerebellar Hypoplasia

Cerebellar abnormalities affect many neurocognitive functions such as language and cognitive‐emotional and are also associated with neurodevelopmental disorders, as well as the cerebellum is associated with motor functions, so its damage can lead to ataxia [40]. Cerebellar hypoplasia is a defect in which the cerebellum is reduced in size but retains its shape [41]. SPG63/AMPD2 patients have pontine cerebellar hypoplasia [42], SPG66/ARSI and SPG67/PGAP1 patients have borderline intelligence with cerebellar hypoplasia [43, 44], and SPG88/KPNA3 patients have mental retardation with cerebellar and brainstem hypoplasia [45].

Cerebellar atrophy represents another manifestation of cerebellar abnormalities, comprising 12.35% of HSP subtypes, accompanied by other symptoms. For example, mental retardation in SPG15/ZFYVE26 and SPG46/GBA2 [46], cognitive impairment and epilepsy in SPG35/FA2H and SPG84/PI4KA [47, 48]. Severe cases may also be accompanied by physical anomalies, such as scoliosis and bowed feet in patients with SPG7/PGN and SPG44/GJC2 [49, 50], and visual loss in SPG79/UCHL1 patients [51].

### 2.4 Ventricular Enlargement

Ventricular enlargement is usually associated with hydrocephalus or cerebral atrophy. In the former condition, the enlarged ventricles are due to increased pressure of cerebrospinal fluid in the skull, which in turn compresses the ventricles and surrounding nerve tissue [52]. In the latter condition, the enlarged ventricles are caused by an abnormal decrease in brain cells and shrinkage of brain tissue due to aging, brain injury, neurological disease, or infection [53]. Both of these causes can lead to enlarged ventricles in patients with HSP, for example, SPG1/L1CAM has enlarged ventricles due to hydrocephalus [54]. A majority of SPG61/ARL6IP1 patients show mild to severe enlargement of the lateral ventricles and of the cerebral sulci, in addition to a thin corpus callosum and diffuse white matter hyperintensity [55]. More patients have mild ventricular enlargement caused by non‐hydrocephalus causes, e.g. SPG47/AP4B1, SPG50/AP4M1, SPG51/AP4E1 [23, 26, 56]. Importantly, recent studies highlight the importance of precisely regulated neuroepithelial cell fate for congenital hydrocephalus [57, 58], indicating neurodevelopmental mechanism of ventricular enlargement.

Collectively, clinical investigations reveal abnormalities associated with altered neurodevelopment in HSP diseases. These neurodevelopmental abnormalities can vary depending on the specific gene mutation involved and the individual's unique genetic background.

## 3 Expression Trajectories of HSP Genes in Developing Brains

**FIGURE 2:**
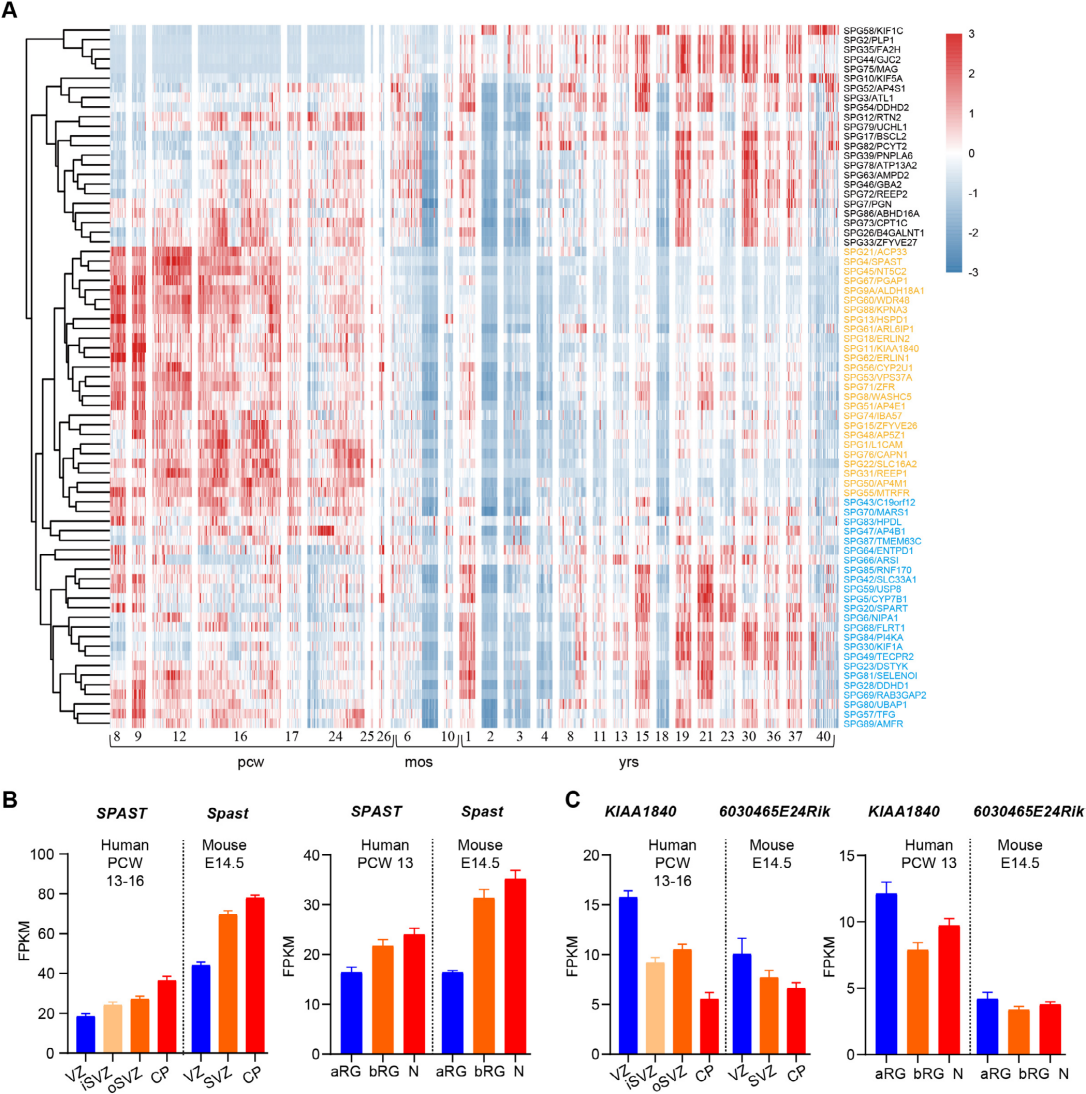
mRNA expression profiles of HSP‐related genes across different developmental stage of brain. (A) A heatmap was utilized to illustrate the mRNA expression levels of 73 HSP‐associated genes in the cortex of the human brain across diverse time periods. (B, C) Mean Fragments Per Kilobase of exon model per Million mapped fragments (FPKM) values of *SPG4/SPAST* and *Spg4*/*Spast* (B), or *SPG11 KIAA1840* and *Spg11/6030465E24Rik* (C) mRNA expression in germinal neocortical zones of fetal human neocortical tissue post‐conception weeks (PCW) 13–16 and embryonic mouse at E14.5. aRG, apical radial glial; bRG, basal radial glial; CP, cortical plate; iSVZ, inner subventricular zone; N, neuron; oSVZ, outer subventricular zone; SVZ, subventricular zone; VZ, ventricular zone.

To better track the expression profiles of the genes responsible for HSP, we investigate gene expression of 73 HSP causing genes in the cortex across pre‐natal and postnatal periods from BrainSpan data (http://www.brainspan.org/). We summarized the expression profiles and found that 23/73 genes have higher expression in late developmental stage (after 6 months of gestation) and after birth, whereas 50/73 genes have higher expression in early development (before 26 weeks of gestation). Among these 50 genes, 26 genes (highlighted in yellow in Figure 2A) exhibit high level during embryonic periods, with their expression subsequently decreasing after birth. Conversely, the remaining 24 genes (highlighted in blue in Figure 2A) display minimal differences in expression between the embryonic and adult periods. Genes that are highly expressed during the embryonic period are prone to being associated with developmental disorders. For example, genes including *SPG1/L1CAM*, *SPG47/AP4B1*, *SPG50/AP4M1*, *SPG51/AP4E1*, and *SPG61*/*ARL6IP*, that caused ventricular enlargement were highly expressed during embryonic period. All of the genes associated with microcephaly or cerebellar hypoplasia, except for AMPD2/SPG63, were highly expressed during the embryonic period. In addition, 63.15% of the genes associated with agenesis of corpus callosum were highly expressed during embryonic period. However, only 46.67% of the genes related to cerebellar atrophy were highly expressed during the embryonic period, with a larger proportion of genes being highly expressed after birth. This may be due to the fact that the cerebellum is one of the last brain structures to achieve maturity [59].

To investigate the expression profile of HSP genes during cortical development, we used the published database GSE38805 [60] and GSE65000 [61] to monitor the transcriptome expression profiles of SPG4/SPAST and SPG11/KIAA1840, which are the primary causes of AD HSP and AR HSP, respectively, across various regions of embryonic brain tissues in humans and mice. *SPG11* showed higher expression in the ventricular zone (VZ) than in cortical plate (CP) in both the human and mouse cortex, whereas *SPG4* showed higher expression in the CP compared with VZ. Consistently, *SPG11* is highly enriched in the apical radial glial, whereas *SPG4* is highly enriched in neurons (Figure 2B,C). These results suggest that the genes involved in AR HSP are more likely to be correlated with progenitor cells and early neurodevelopment.

## 4 HSP Proteins Play Roles in Brain Development

**FIGURE 3:**
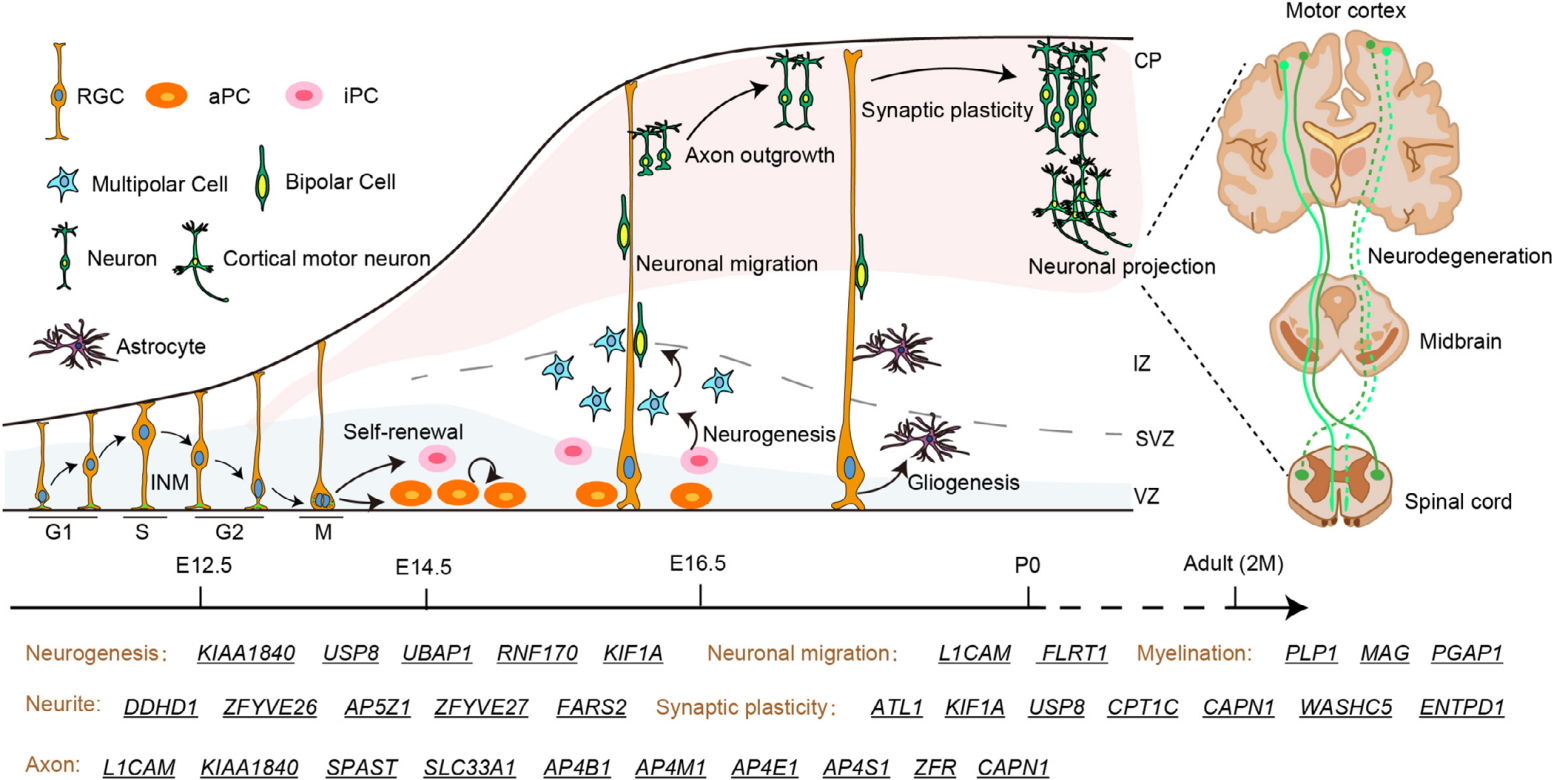
Schematic illustration of HSP proteins regulating different brain development processes. HSP proteins function in neurogenesis, neuronal migration, and neuronal differentiation including axonal and neurite outgrowth, dendritic spines and neural plasticity, and myelination. CP, cortical plate; IZ, intermediate zone; SVZ, subventricular zone; VZ, ventricular zone.

Brain development is a highly ordered process that is subject to precise temporal and spatial regulation. Neural stem cells possess self‐renewal capabilities and multilineage differentiation potential. Under specific conditions, neural stem cells can differentiate into neurons, oligodendrocytes, and astrocytes [62]. Immature neurons migrate from their origin to specific sites where they are organized into layers, nuclei, and ganglia of neuronal cell bodies. Subsequently, these neurons sprout axons and dendrites, which can extend for several centimeters, to establish synaptic connections with target neurons or other cellular entities [63] (Figure 3).

### 4.1 Neurogenesis

During brain development, neural stem and progenitor cells generate neurons via a process called neurogenesis. Neurogenesis involves proliferation and differentiation and linked with cell cycle regulation. Several fundamental cellular biological processes play crucial roles in determining neurogenesis, including cell polarity, mitotic spindle and cleavage plane orientation, symmetric versus asymmetric cell division, cell cycle regulation, INM, and progenitor cell delamination [64].

There is growing evidence of HSP proteins function in the different developmental process of neurogenesis which is more extensive studied in SPATACSIN/SPG11. Study of iPSC‐derived neural progenitor cells (NPCs) from SPG11 patients showed a decrease in S‐phase and G2/M‐phases and a reduction in neuronal cell proliferation. Interestingly, the developmental defect of SPG11‐NPCs was found to be caused by dysregulated GSK3β signaling. Importantly, this defect can be mitigated by GSK3β inhibitors, providing a promising basis for the development of early interventions for neurodegenerative diseases [65]. A follow‐up study has unveiled that the observed proliferation deficiencies in cerebral organoids are attributed to an elevated asymmetric division rate of NPCs derived from HSP patients [66]. Consistently, these neurodevelopmental abnormalities were mitigated in both experimental models following the administration of Tideglusib, a specific GSK3β inhibitor [65, 66]. Consistent with KIF1A‐associated neurological disorder in children, *Kif1a* null mouse showed reduced brain size [67]. An RNAi screen of kinesin genes identified *Kif1a*, as the motor for basally directed nuclear movement of radial glial cells [68]. In addition, *Kif1a* RNAi results in a decrease in neurogenic divisions and blocks multipolar‐bipolar transition in neurons [68, 69]. Similarly, recent study indicate KIF1A serve together with KIF3B in controlling the balance between symmetric and asymmetric divisions, and, in turn, the timing and extent of neurogenesis in the rat brains [70]. Ubiquitin specific peptidase 8 (USP8/SPG59) maintains embryonic stem cell stemness via deubiquitination of ectopic P‐granules 5 autophagy tethering factor (EPG5) [71]. Ubiquitin associated protein 1 (UBAP1/SPG80) deficiency disrupts apical junctions and polarity of RGCs, leading to cortical neurogenesis defects and prenatal ventriculomegaly [58]. Knockdown of RNF170 (SPG85) results in morphological abnormalities, impaired neurogenesis, and motor neuron defects [48, 72].

### 4.2 Neuronal Differentiation

Neurons are highly polarized cells with distinct subcellular compartments, including dendritic arbors and an axon. Axons are the primary transmission lines of the nervous system, and as bundles they form nerves [73]. Axon carry electrical or chemical signals away from nerve cells, which allows them to send messages to nerve, gland, or muscle cells [74]. Voluntary movement in humans relies on the pyramidal motor system that extends from the cerebral motor cortex to neuromuscular junctions innervating skeletal muscle [73]. HSPs are a large, genetically diverse group of inherited neurologic disorders characterized by a length‐dependent distal axonopathy of the corticospinal tracts [75]. During the developmental process, any error in the process of neurons can potentially cause axon defects. In addition, type‐specific dendrite morphology is a hallmark of the neuron and has important functional implications in determining what signals a neuron receives and how these signals are integrated [76].

#### 4.2.1 Axon and Neurite Outgrowth

SPG1/L1CAM, the gene encoding the transmembrane multifunctional neuronal adhesion molecule L1, is associated with the neurodevelopmental disorders and different process of brain development including differentiation of adult‐born hippocampal neurons, axonal, and dendritic arborization and neuronal migration [77, 78, 79, 80]. Neurons differentiated from iPSC of SPG4/SPAST patients exhibit a series of axonal defects, including decreased levels of stable microtubules, lower peroxisome transport speed as a consequence of reduced microtubule‐dependent transport, reduced number of peroxidases, and higher axonal swelling density [81]. The functional importance of SPG11/SPATACSIN in neurodevelopment was confirmed through zebrafish embryo studies. These studies revealed that SPG11 morphant embryos exhibited developmental defects and central nervous system abnormalities, with axon pathway formation being particularly disrupted [82]. Knockout of SPG11 has been shown to impair lipid clearance in lysosomes, leading to neuronal lipid accumulation [83], as well as to disrupt cholesterol transport and calcium homeostasis [84]. Depletion of SPG42/SLC33A1 affects axonal growth and leads to poor spinal cord growth [85]. *Slc33a1* knockdown zebrafish exhibited defects in morphology and axon outgrowth, which could be rescued by human wild‐type *Slc33a1* mRNA [86]. Analyses and simulations suggest that sulcus formation in the absence of SPG68/FLRT1/3 results from reduced intercellular adhesion, increased neuron migration, and clustering in the cortical plate [87]. In addition, the FLRTs (FLRT1–3) are regulators of early embryonic, vascular, and neural development [88]. In *Caenorhabditis elegans* , loss of SPG76/CAPN1 leads to neuronal and axonal dysfunction and degeneration [89], whereas loss of SPG71/ZRF results in axon midline crossing, axon defasciculation, and cord commissures [90].

A deficiency in the adaptor protein complex 4 (AP‐4), such as SPG47/AP4B1, SPG50/AP4M1, SPG51/AP4E1, and SPG52/AP4S1, can trigger the onset of HSP in childhood, revealing key developmental mechanisms. Neurons from AP‐4‐deficient mice exhibit axon‐specific defects including reduced length and reduced branching [91]. Similarly, iPSC‐derived neurons from patients with AP‐4 deficiency also have reduced neurite length and reduced branching at an early stage of development [92]. SPG28/DDHD1 and SPG54/DDHD2 are both intracellular phospholipases A1 and hydrolyze phosphatidic acid in vitro. DDHD1, but Not DDHD2, suppresses neurite outgrowth in SH‐SY5Y and PC12 cells by regulating protein transport from recycling endosomes [93]. SPG15/ZFYVE26 and SPG48/AP5Z1 encode two proteins in the same complex, respectively. Their mutations result in mitochondrial dysfunction of iPSC derived glutamatergic and midbrain dopaminergic neurons in patients, leading to a significant reduction in the neurite number, length, and branching [94]. ZFYVE27 is a member of FYVE family, which is implicated in the formation of neurite extensions by promoting directional membrane trafficking in neurons through its own oligomerization [95]. SPG77/FARS2 deficiency leads to delayed neurite outgrowth of primary neurons in mice, followed by neuronal apoptosis [96].

#### 4.2.2 Dendritic Spines and Neural Plasticity

It has been shown that atlastin GTPase 1 (ATL1/SPG3A) controls neuronal dendritic spine formation by regulating tubular endoplasmic reticulum (ER) formation in conjunction with valosin‐containing protein and influencing the efficiency of protein synthesis [97, 98]. In addition, ATL1 binds to numerous HSP gene products involving shaping the tubular ER network including SPG4/SPATIN, SPG31/REEP1, SPG72/REEP2, and SPG12/RTN2 [99, 100, 101]. Meanwhile, ATL1 regulates dendritic morphogenesis through its GTPase activity. In cultured cortical neurons, overexpression of ATL1 increased dendritic growth and branching. However, this beneficial effect is absent in the HSP‐related R217Q mutant, which lacks the necessary GTPase activity [102]. WASH complex subunit 5 (WASHC5/SPG8) regulates structural plasticity in cortical neurons by modulating actin polymerization. Its absence reduces dendritic branching and synapse formation in neurons [103]. In *Caenorhabditis elegans* neurons, KIF1A (SPG30)/UNC‐104 transports ATG‐9 to regulate neurodevelopment and autophagy at synapses [104]. Auto‐inhibition of a neuronal kinesin KIF1A regulates the size and density of synapses [105]. SPG59/USP8 deubiquitinates SHANK3 to control synapse density and SHANK3 activity‐dependent protein levels [106]. Deficiency of carnitine palmitoyl transferase 1C (CPT1C/SPG73), a neuron‐specific interacting protein involved in AMPA receptor synthesis and trafficking, disrupts hippocampal dendritic spine maturation and long‐term synaptic plasticity and reduces cortical γ‐oscillations [107]. Loss of calpain 1 (CAPN1/SPG76) does not affect dendritic branching patterns but leads to fewer mature dendritic spines and more naive dendritic spines, resulting in impaired synaptic transmission [108]. Differently, ectonucleoside triphosphate diphosphohydrolase‐1 (ENTPD1/SPG64) deletion leads to increased hippocampal neurogenesis as well as neuronal dendritic spines [109].

#### 4.2.3 Myelination

Proteolipid protein 1 (PLP1/SPG2) is associated with late gestational myelin formation in humans and is one of the major genes encoding myelin proteins. PLP‐deficient mice develop normally and have normal amounts of myelin but become physically fragile, a phenomenon also found in patients [110]. Defects in post‐GPI attachment to proteins inositol deacylase‐1 (PGAP1/SPG67) may delay myelin formation [111]. In addition, myelin‐associated glycoprotein (MAG/SPG75) is also associated with myelination, but its function and mechanism in the central nervous system are not yet clear.

Taken together, different HSP proteins are implicated in different processes of brain development which may ultimately lead to structural and functional abnormalities of cortical motor neurons.

## 5 Limitations

Most studies revealed the clinical manifestation of HSP patients with certain time period, it remained unclear whether brain abnormality such as atrophy of the brain or cerebellum is result from developmental or degenerative condition.

To distinguish between the role of neural development and degeneration, it is necessary to observe and study the entire life cycle of patients. Since most HSPs are reported with early (neonatal, infancy, or childhood) onset, HSP causing genes are more likely to play pivotal roles in growth and development [112]. However, there is still limited research on the developmental mechanisms of related genes. In addition, conventional knock out of some HSP genes such as *Vps37a* and *Ubap1* result in embryonic lethal in mice, which limit the study of gene function on brain development.

## 6 Concluding and Prospects

The accepted dogma divides neurodegenerative and neurodevelopmental diseases apart. However, emerging evidence indicates that neurogenetic conditions associated primarily with neurodegeneration also affect neurodevelopment [113]. Here we summary the clinical manifestation, gene expression, and protein function of HSP. We found disruption of some HSP proteins led to global neurodevelopmental delay, microcephaly, and other developmental disorders (Figure 1). These phenotypes were supported by the fact that HSP genes play roles in the proliferation and differentiation of neural stem cells, as well as in the migration and maturation of neurons (Figure 3). In addition, the early expression pattern of some HSP genes in the human brain further supports their role in neural development (Figure 2). Most genes are expressed abundantly during critical developmental stages after conception. Due to the role of HSP genes in neural development, early identification and intervention may help mitigate disease progression and improve patients' quality of life. By analyzing the clinical manifestations and gene expression patterns of patients, possible subtypes of HSP can be inferred and targeted screening can be carried out. Consequently, this implies that we have the potential to assess the likelihood of neurodegenerative diseases via early developmental diagnosis, potentially even delaying the onset and progression of these diseases through timely intervention.

Neurodegenerative diseases are usually characterized by progressive neuronal loss and dysfunction, whereas neurodevelopmental diseases involve abnormalities in neuronal development. For example, cerebellar hypoplasia is a condition in which the cerebellum fails to reach its normal size during development, whereas cerebellar atrophy is a condition in which the cerebellum decreases in size due to neuronal loss. Both conditions reflect vulnerabilities in the development and maintenance of the nervous system. A typically biphasic disease course of late‐onset neurodegeneration occurred on the background of a neurodevelopmental disorder [114]. The close connection with neurodegenerative disorders highlights the relevance of research into rare early‐onset neurodevelopmental conditions for much more common, age‐related human diseases. Although HSP has traditionally been recognized as a neurodegenerative disease, there is growing evidence that the pathogenesis of HSP involves abnormalities in neurodevelopment. The fact that most patients with HSP present with symptoms in infancy or even neonatally, emphasizes the importance of recognizing HSP as a “neurodevelopmental‐related disorder”.

Since HSP is a neurogenetic disorder, mutation of proteins happens at the very beginning of organ development. Some HSP proteins have been proved to play import roles in different process of normal brain development. Neurogenesis is active during the embryonic period, as it is responsible for producing new neurons. The decreased proliferation of NPC results in a smaller population of neurons, potentially impacting the reserve available for replacing degenerated or diseased nerve cells in later stages, ultimately causing neurodegenerative symptoms. Neuronal migration, neurite outgrowth, and synaptic plasticity all contribute to maintain neural network connections. This abnormal development might be partly responsible for the selective vulnerability of neurons in degenerative diseases [113]. Finally, HSP proteins have been implicated in signaling pathways associated with neurodevelopment, such as Wnt and BMP signaling [65, 75]. Understanding the roles and signaling pathways of these proteins during development may ‐offer new drug targets for therapy and predictors of susceptibility useful for prevention of HSP.

HSPs can be diagnosed early through genetic screening, measures of growth, including height, weight, and body mass index, between child and adolescent carriers of HSP associated mutations and control peers will help to identify the developmental trajectory of HSPs. In addition, as the formation of the Sylvian fissure appears early in utero [115], various 3D reconstruction of sulcal features can then be analyzed to explore the genetic interplay with neurodevelopment. Ultimately, examining the roles of HSP genes using spatiotemporal specific genetic knockout mice and organoids derived from HSP patients is crucial for dissecting the consequences of deletion/mutation on early neural development.

Overall, as HSP is a phenotypically and genotypically diverse group of monogenic diseases, collaborative and multidisciplinary efforts are required to provide systemic insights into both the clinical manifestation and pathogenesis of HSP, which will benefit for developing effective therapeutic strategies.

## Author Contributions

Manuscript conception and design: D.X. and Y.Z.; literature search and data analysis: D.X., Y.Z., Y.S., and D.L.; manuscript writing: Y.Z. and D.X. All authors approved the final version of this manuscript.

## Conflicts of Interest

The authors declare no conflicts of interest.

## Supporting information


Table S1.

Table S2.

## Data Availability

The data that support the findings of this study are available from the corresponding author upon reasonable request.
