# Supplementary material for: Neurodevelopmental Implications Underpinning Hereditary Spastic Paraplegia
Source: CNS Neurosci Ther. 2025 Feb 11;31(2):e70260. doi: 10.1111/cns.70260 (PMC11811889; doi:10.1111/cns.70260)
Supplement: Supplementary file 1 — Table S1. [file CNS-31-e70260-s002.docx]

| **Table S1 HSP subtypes, chromosomal localization, mode of inheritance and clinical manifestations** | | | | | | |
| --- | --- | --- | --- | --- | --- | --- |
| **Gene** | **subtype** | **Chromosomal localization** | **inheritance** | **Clinical manifestations** | **Other characteristic manifestations** |  |
| L1CAM | SPG1 | Xq28 | X-linked | Complicated | Agenesis of the corpus callosum, mental retardation, thumb adduction, ventricular, enlargement and hydrocephalus [^1^](#_ENREF_1) |  |
| PLP1 | SPG2 | Xq22 | X-linked | Pure or complicated | Mental retardation, ataxia, white matter degeneration, epilepsy, multiple neuropathies [^2^](#_ENREF_2) |  |
| ATL1 | SPG3A | 14q22 | AR/AD | Pure or complicated | Mental retardation, dementia, epilepsy, ataxia [^3^](#_ENREF_3) |  |
| SPAST | SPG4 | 2p22 | AD | Pure or complicated | Cognitive impairment, epilepsy, ataxia, arched foot, posterior fossa deformity, white matter degeneration [^4^](#_ENREF_4)^,^[^5^](#_ENREF_5) |  |
| CYP7B1 | SPG5 | 8q12 | AR | Pure or complicated | Deep sensory impairment and white matter degeneration [^6^](#_ENREF_6) |  |
| NIPA1 | SPG6 | 15q11 | AD | Pure or complicated | Epilepsy, peripheral neuropathy, amyotrophic lateral sclerosis, memory deficits, cognitive impairment [^7^](#_ENREF_7) |  |
| PGN | SPG7 | 16q24 | AR | Pure or complicated | Cerebellar atrophy, polyneuropathy, optic nerve atrophy [^8^](#_ENREF_8) |  |
| WASHC5 | SPG8 | 8q24 | AD | Pure | [^9^](#_ENREF_9) |  |
| ALDH18A1 | SPG9 | 10q24 | AR/AD | Pure or complicated | Pseudobulbar palsy, short stature, microcephaly, facial deformities [^10^](#_ENREF_10) |  |
| **Gene** | **subtype** | **Chromosomal localization** | **inheritance** | **Clinical manifestations** | **Other characteristic manifestations** |  |
| KIF5A | SPG10 | 12q13 | AD | Pure or complicated | Cognitive impairment, polyneuropathy, autonomic dysfunction, Parkinson's syndrome, deafness [^11^](#_ENREF_11) |  |
| KIAA1840 | SPG11 | 15q13 | AR | Complicated | Mental retardation, cerebellar atrophy, Parkinson's syndrome, epilepsy, multiple neuropathies, thin corpus callosum, abnormal eye signs, macular degeneration, multiple neuropathies [^12^](#_ENREF_12) |  |
| RTN2 | SPG12 | 19q13 | AD | Pure | Visual abnormalities, sphincter disturbances, seizures [^13^](#_ENREF_13) |  |
| HSPD1 | SPG13 | 2q33 | AD | Pure | Speech and motor delay, mild ataxia [^14^](#_ENREF_14) |  |
| unknow | SPG14 | 3q27-q28 | AR | Complicated | Mental retardation, polyneuropathy [^15^](#_ENREF_15) |  |
| ZFYVE26 | SPG15 | 14q24 | AR | Complicated | Motor and/or speech delay, learning disabilities, cerebellar ataxia and dystonia [^16^](#_ENREF_16) |  |
| unknown | SPG16 | Xq11 | X-linked | Pure or complicated | Mental retardation, aphasia, vision loss, nystagmus [^17^](#_ENREF_17) |  |
| BSCL2 | SPG17 | 11q12 | AD | Complicated | Sensory disturbance [^18^](#_ENREF_18) |  |
| ERLIN2 | SPG18 | 8p12 | AD/AR | Pure or complicated | Epilepsy, mental retardation, polyarticular contracture [^19^](#_ENREF_19) |  |
| unknow | SPG19 | 9q33 | AD | Pure | [^20^](#_ENREF_20) |  |
| **Gene** | **subtype** | **Chromosomal localization** | **inheritance** | **Clinical manifestations** | **Other characteristic manifestations** |  |
| SPART | SPG20 | 13q13 | AR | Complicated | Mental retardation, dysarthria, upper extremity Spasticity, white matter abnormalities [^21^](#_ENREF_21) |  |
| ACP33 | SPG21 | 15q22 | AR | Complicated | Dysarthria, thin corpus callosum, dementia, brain white matter degeneration, extrapyramidal signs [^22^](#_ENREF_22) |  |
| SLC16A2 | SPG22 | Xq13 | X-linked | Complicated | Mental retardation, ataxia, muscular dystrophy Ataxia, myasthenia gravis, nystagmus [^23^](#_ENREF_23) |  |
| DSTYK | SPG23 | 1q32 | AR | Complicated | Skin pigmentation abnormalities [^24^](#_ENREF_24) |  |
| unknow | SPG24 | 13q14 | AR | Pure | [^25^](#_ENREF_25) |  |
| unknow | SPG25 | 6q23 | AR | Complicated | Herniated disc, polyneuropathy [^26^](#_ENREF_26) |  |
| B4GALNT1 | SPG26 | 12q13 | AR | Complicated | Mental retardation, cerebral white matter degeneration, distal muscle atrophy [^27^](#_ENREF_27) |  |
| unknow | SPG27 | 10q22 | AR | Pure | [^28^](#_ENREF_28) |  |
| DDHD1 | SPG28 | 14q22 | AR | Pure or complicated | Weakness in the lower extremities and bilateral Babinski reflexes [^29^](#_ENREF_29) |  |
| unknow | SPG29 | 1p31 | AD | Complicated | Hyperbilirubinemia, hearing loss, esophageal hiatal hernia [^30^](#_ENREF_30) |  |
| **Gene** | **subtype** | **Chromosomal localization** | **inheritance** | **Clinical manifestations** | **Other characteristic manifestations** |  |
| KIF1A | SPG30 | 2q37 | AR/AD | Pure or complicated | Microcephaly[^31^](#_ENREF_31), pronounced mental retardation |  |
| REEP1 | SPG31 | 2p11 | AD | Pure or complicated | Polyneuropathy, ataxia, carpal tunnel carpal tunnel syndrome [^32^](#_ENREF_32) |  |
| unknow | SPG32 | 14q12 | AR | Complicated | Mental retardation, thin corpus callosum [^33^](#_ENREF_33) |  |
| ZFYVE27 | SPG33 | 10q24 | AD | Pure | [^34^](#_ENREF_34) |  |
| unknow | SPG34 | Xq25 | X-linked | Pure | [^35^](#_ENREF_35) |  |
| FA2H | SPG35 | 16q23 | AR | Pure or complicated | Cognitive impairment, cerebellar atrophy, epilepsy, cerebral leukodystrophy, optic atrophy Cerebral leukodystrophy, optic atrophy [^36^](#_ENREF_36) |  |
| unknow | SPG36 | 12q23 | AD | Complicated | Polyneuropathy [^37^](#_ENREF_37) |  |
| unknow | SPG37 | 8p21 | AD | Pure | [^38^](#_ENREF_38) |  |
| unknow | SPG38 | 4p16 | AD | Complicated | Hand and foot muscular dystrophy, polyneuropathy [^39^](#_ENREF_39) |  |
| PNPLA6 | SPG39 | 19p13 | AR | Complicated | Distal muscular atrophy, polyneuropathy, cerebellar atrophy [^40^](#_ENREF_40) |  |
| unknow | SPG41 | 11p14 | AD | Pure | [^41^](#_ENREF_41) |  |
| SLC33A1/AT-1 | SPG42 | 3q25 | AD | Pure | Development delay [^42^](#_ENREF_42) |  |
| **Gene** | **subtype** | **Chromosomal localization** | **inheritance** | **Clinical manifestations** | **Other characteristic manifestations** |  |
| C19ORF12 | SPG43 | 19q12 | AR | Complicated | Joint contractures and proximal weakness of the lower extremities [^43^](#_ENREF_43) |  |
| GJC2 | SPG44 | 1q41 | AR | Complicated | Cognitive impairment, cerebellar atrophy, dysarthria, thin corpus callosum, scoliosis, high arched feet, strabismus [^44^](#_ENREF_44) |  |
| NT5C2 | SPG45 | 10q24 | AR | Complicated | Mental retardation, lower limb joint contractures, clubfoot, optic nerve atrophy and thin corpus callosum [^45^](#_ENREF_45) |  |
| GBA2 | SPG46 | 9p13 | AR | Complicated | Cognitive impairment, thin corpus callosum, cataracts, cerebellar atrophy [^46^](#_ENREF_46) |  |
| AP4B1 | SPG47 | 1p13 | AR | Complicated | Mental retardation, epilepsy, thin corpus callosum, cerebral white matter degeneration, microcephaly, ventricular enlargement and joint laxity [^47^](#_ENREF_47) |  |
| AP5Z1 | SPG48 | 7p22 | AR | Pure or complicated | Polyneuropathy, ataxia, cerebral white Mass degeneration, spinal cord high signal, hypoplastic corpus callosum [^48^](#_ENREF_48) |  |
| TECPR2 | SPG49 | 14q32 | AR | Complicated | Mental retardation, gastroesophageal reflux, thin corpus callosum, malformation, microcephaly [^49^](#_ENREF_49) |  |
| **Gene** | **subtype** | **Chromosomal localization** | **inheritance** | **Clinical manifestations** | **Other characteristic manifestations** |  |
| AP4M1 | SPG50 | 7q22 | AR | Complicated | Mental retardation, bunions, microcephaly, ventricular enlargement, white matter degeneration, cerebellar atrophy, hypoplastic corpus callosum [^50^](#_ENREF_50) |  |
| AP4E1 | SPG51 | 15q21 | AR | Complicated | Mental retardation, microcephaly, ventricular enlargement, cerebral white matter degeneration, cerebellar atrophy [^51-53^](#_ENREF_51) |  |
| AP4S1 | SPG52 | 14q12 | AR | Complicated | Short stature, delayed speech, mental retardation [^54^](#_ENREF_54) |  |
| VPS37A | SPG53 | 8p22 | AR | Complicated | Mental retardation, hirsutism, kyphosis [^55^](#_ENREF_55) |  |
| DDHD2 | SPG54 | 8p11 | AR | Complicated | Mental retardation, strabismus, dysarthria, epilepsy, optic nerve hypoplasia, lateral torsion foot, thin corpus callosum, cerebral white matter degeneration [^56^](#_ENREF_56) |  |
| MTRFR | SPG55 | 12q24 | AR | Complicated | Optic nerve atrophy, clubfoot, hypoplastic corpus callosum  [^57^](#_ENREF_57) |  |
| CYP2U1 | SPG56 | 4q25 | AR | Pure or complicated | Macular degeneration, cognitive decline, dystonia Impairment, thin corpus callosum, cerebral white matter degeneration [^58^](#_ENREF_58) |  |
| **Gene** | **subtype** | **Chromosomal localization** | **inheritance** | **Clinical manifestations** | **Other characteristic manifestations** |  |
| TFG | SPG57 | 3q12 | AR | Complicated | Optic nerve atrophy, polyneuropathy [^59^](#_ENREF_59) |  |
| KIF1C | SPG58 | 17p13 | AR/AD | Pure or complicated | Mental retardation, deafness, ataxia, chorea, short stature, bowed feet, brain white matter degeneration [^60^](#_ENREF_60) |  |
| USP8 | SPG59 | 15q21 | AR | Complicated | Mild cognitive impairment [^61^](#_ENREF_61), neurodevelopment delay[^62^](#_ENREF_62) |  |
| WDR48 | SPG60 | 3p22 | AR | Complicated | Nystagmus, mild cognitive impairment [^61^](#_ENREF_61) |  |
| ARL6IP1 | SPG61 | 16p12 | AR | Complicated | Intellectual disability, ventricular enlargement, agenesis of the corpus callosum [^63^](#_ENREF_63) |  |
| ERLIN1 | SPG62 | 10q24 | AR | Pure | Intellectual disability, epilepsy[^64^](#_ENREF_64) |  |
| AMPD2 | SPG63 | 1p13 | AR | Complicated | Thin corpus callosum, microcephaly, cerebellar hypoplasia, developmental delay, spasticity[^65^](#_ENREF_65) |  |
| ENTPD1 | SPG64 | 10q24 | AR | Complicated | Cognitive impairment, dysarthria/anarthria, dystonia and areflexia[^66^](#_ENREF_66) |  |
| NT5C2 | SPG65 | 10q24 | AR | Pure or complicated | Thin corpus callosum, arched feet [^61^](#_ENREF_61) |  |
| ARSI | SPG66 | 5q32 | AR | Complicated | Clubfoot, borderline intelligence, corpus callosum and cerebellar hypoplasia [^61^](#_ENREF_61) |  |
| **Gene** | **subtype** | **Chromosomal localization** | **inheritance** | **Clinical manifestations** | **Other characteristic manifestations** |  |
| PGAP1 | SPG67 | 2q33 | AR | Complicated | Borderline intelligence, corpus callosum hypoplasia, cerebellar hypoplasia, tremor [^61^](#_ENREF_61); severely delayed myelination and psychomotor retardation[^67^](#_ENREF_67);  global developmental delay and encephalopathy [^68^](#_ENREF_68). |  |
| FLRT1 | SPG68 | 11q13 | AR | Complicated | Optic nerve atrophy, polyneuropathy, muscular atrophy, foot drop [^69^](#_ENREF_69) |  |
| RAB3GAP2 | SPG69 | 1q41 | AR | Complicated | Mental retardation, deafness, cataracts [^70^](#_ENREF_70) |  |
| MARS1 | SPG70 | 12q13 | AR | Complicated | Neurodevelopmental delay, corpus callosum hypoplasia, anemia, hepatomegaly, feeding difficulties[^71^](#_ENREF_71) |  |
| ZFR | SPG71 | 5p13 | AR | Complicated | [^72^](#_ENREF_72) |  |
| REEP2 | SPG72 | 5q31 | AR/AD | Pure | [^73^](#_ENREF_73) |  |
| CPT1C | SPG73 | 19q13 | AD | Pure | [^74^](#_ENREF_74) |  |
| IBA57 | SPG74 | 1q42 | AR | Complicated | Optic nerve atrophy, bowed foot, distal muscular atrophy  [^75^](#_ENREF_75) |  |
| **Gene** | **subtype** | **Chromosomal localization** | **inheritance** | **Clinical manifestations** | **Other characteristic manifestations** |  |
| MAG | SPG75 | 19q13 | AR | Complicated | Mental retardation, ataxia, myasthenia gravis, peripheral neuropathy [^61^](#_ENREF_61) |  |
| CAPN1 | SPG76 | 11q12 | AR | Complicated | Dysarthria, bowed feet, and ataxia, polyneuropathy [^76^](#_ENREF_76) |  |
| FARS2 | SPG77 | 6p25 | AR | Pure | [^77^](#_ENREF_77) |  |
| ATP13A2 | SPG78 | 1p36 | AR | Complicated | Polyneuropathy, Parkinson's syndrome, ataxia, cognitive decline [^78^](#_ENREF_78) |  |
| UCHL1 | SPG79 | 4p13 | AR | Complicated | Vision loss, head tremor, optic nerve atrophy shrinkage, cerebellar atrophy [^79^](#_ENREF_79) |  |
| UBAP1 | SPG80 | 9p13 | AD | Pure | [^80^](#_ENREF_80) |  |
| SELENOI | SPG81 | 2p23 | AR | Complicated | Microcephaly, developmental delay, epilepsy, retinitis pigmentosa, leukoencephalopathy retinitis pigmentosa, cerebral white matter degeneration [^81^](#_ENREF_81) |  |
| PCYT2 | SPG82 | 17q25 | AR | Complicated | Mental retardation, epilepsy, optic nerve atrophy [^82^](#_ENREF_82) |  |
| HPDL | SPG83 | 1p34 | AR | Pure | Microcephaly, brain atrophy, epilepsy, and severe intellectual and motor disability [^83^](#_ENREF_83) |  |
| **Gene** | **subtype** | **Chromosomal localization** | **inheritance** | **Clinical manifestations** | **Other characteristic manifestations** |  |
| PI4KA | SPG84 | 22q11 | AR | Complicated | Limb spasticity, developmental delay, thin corpus callosum and progressive cerebellar atrophy, intellectual disability, seizures, ataxia, nystagmus [^84^](#_ENREF_84) |  |
| RNF170 | SPG85 | 8p11 | AR | Complicated | Optic nerve atrophy, ataxia [^85^](#_ENREF_85) |  |
| ABHD16A | SPG86 | 6p21 | AR | Complicated | Mental retardation, progressive spasticity, corpus callosum abnormalities, white matter abnormalities [^86^](#_ENREF_86) |  |
| TMEM63C | SPG87 | 14q24 | AR | Complicated | Mental retardation, microcephaly [^87^](#_ENREF_87) |  |
| KPNA3 | SPG88 | 13q14 | AD | Complicated | Motor retardation, mental retardation, hypoplastic cerebellum and brainstem [^88^](#_ENREF_88) |  |
| AMFR | SPG89 | 16q13 | AR | Pure or complicated | Developmental delay, mild mental retardation, motor dysfunction and progressive spasticity [^89^](#_ENREF_89) |  |
| SPTSSA | SPG90 | 14q13 | AR | Complicated | Progressive movement disorders, variable sensorineural hearing loss, speech/cognitive dysfunction [^90^](#_ENREF_90) |  |
| AD= autosomal dominant; AR= autosomal recessive; X-linked= X-linked recessive | | | | | | |

**References**

1. Marin R, Ley-Martos M, Gutierrez G, et al. Three cases with L1 syndrome and two novel mutations in the L1CAM gene. *Eur J Pediatr.* 2015;174(11):1541-1544.

2. Yao L, Zhu Z, Zhang C, et al. PLP1 gene mutations cause spastic paraplegia type 2 in three families. *Ann Clin Transl Neurol.* 2023;10(3):328-338.

3. Kadnikova V, Rudenskaya G, Stepanova A, et al. Mutational Spectrum of Spast (Spg4) and Atl1 (Spg3a) Genes In Russian Patients With Hereditary Spastic Paraplegia. *Sci Rep.* 2019;9(1):14412.

4. Solowska JM, Baas PW. Hereditary spastic paraplegia SPG4: what is known and not known about the disease. *Brain.* 2015;138(Pt 9):2471-2484.

5. Wang J, Bu W, Zhu M, et al. Novel mutation of SPG4 gene in a Chinese family with hereditary spastic paraplegia: A case report. *World J Clin Cases.* 2023;11(14):3288-3294.

6. Prestsæter S, Koht J, Lamari F, et al. Elevated hydroxycholesterols in Norwegian patients with hereditary spastic paraplegia SPG5. *J Neurol Sci.* 2020;419:117211.

7. Spagnoli C, Schiavoni S, Rizzi S, et al. SPG6 (NIPA1 variant): A report of a case with early-onset complex hereditary spastic paraplegia and brief literature review. *J Clin Neurosci.* 2021;94:281-285.

8. Votsi C, Ververis A, Nicolaou P, et al. A Novel SPG7 Gene Pathogenic Variant in a Cypriot Family With Autosomal Recessive Spastic Ataxia. *Front Genet.* 2021;12:812640.

9. Ginanneschi F, D'Amore A, Barghigiani M, et al. SPG8 mutations in Italian families: clinical data and literature review. *Neurol Sci.* 2020;41(3):699-703.

10. Fischer B, Callewaert B, Schroter P, et al. Severe congenital cutis laxa with cardiovascular manifestations due to homozygous deletions in ALDH18A1. *Mol Genet Metab.* 2014;112(4):310-316.

11. Reid E, Kloos M, Ashley-Koch A, et al. A kinesin heavy chain (KIF5A) mutation in hereditary spastic paraplegia (SPG10). *Am J Hum Genet.* 2002;71(5):1189-1194.

12. Doleckova K, Roth J, Stellmachova J, et al. SPG11: clinical and genetic features of seven Czech patients and literature review. *Neurol Res.* 2022;44(5):379-389.

13. Tian W, Zheng H, Zhu Z, et al. New phenotype of RTN2-related spectrum: Complicated form of spastic paraplegia-12. *Ann Clin Transl Neurol.* 2022;9(8):1108-1115.

14. Svenstrup K, Bross P, Koefoed P, et al. Sequence variants in SPAST, SPG3A and HSPD1 in hereditary spastic paraplegia. *J Neurol Sci.* 2009;284(1-2):90-95.

15. Vazza G, Zortea M, Boaretto F, et al. A new locus for autosomal recessive spastic paraplegia associated with mental retardation and distal motor neuropathy, SPG14, maps to chromosome 3q27-q28. *Am J Hum Genet.* 2000;67(2):504-509.

16. Saffari A, Kellner M, Jordan C, et al. The clinical and molecular spectrum of ZFYVE26-associated hereditary spastic paraplegia: SPG15. *Brain.* 2023;146(5):2003-2015.

17. Tamagaki A, Shima M, Tomita R, et al. Segregation of a pure form of spastic paraplegia and NOR insertion into Xq11.2. *Am J Med Genet.* 2000;94(1):5-8.

18. Ishihara S, Okamoto Y, Tanabe H, et al. Clinical features of inherited neuropathy with BSCL2 mutations in Japan. *J Peripher Nerv Syst.* 2020;25(2):125-131.

19. Chen S, Zou J, He S, et al. More autosomal dominant SPG18 cases than recessive? The first AD-SPG18 pedigree in Chinese and literature review. *Brain Behav.* 2021;11(12):e32395.

20. Valente E, Brancati F, Caputo V, et al. Novel locus for autosomal dominant pure hereditary spastic paraplegia (SPG19) maps to chromosome 9q33-q34. *Ann Neurol.* 2002;51(6):681-685.

21. Tawamie H, Wohlleber E, Uebe S, et al. Recurrent null mutation in SPG20 leads to Troyer syndrome. *Mol Cell Probes.* 2015;29(5): 315-318.

22. Ishiura H, Takahashi Y, Hayashi T, et al. Molecular epidemiology and clinical spectrum of hereditary spastic paraplegia in the Japanese population based on comprehensive mutational analyses. *J Hum Genet.* 2014;59(3):163-172.

23. Schwartz C, May M, Carpenter N, et al. Allan-Herndon-Dudley syndrome and the monocarboxylate transporter 8 (MCT8) gene. *Am J Hum Genet.* 2005;77(1):41-53.

24. Lee JYW, Hsu CK, Michael M, et al. Large Intragenic Deletion in DSTYK Underlies Autosomal-Recessive Complicated Spastic Paraparesis, SPG23. *Am J Hum Genet.* 2017;100(2):364-370.

25. Hodgkinson C, Bohlega S, Abu-Amero S, et al. A novel form of autosomal recessive pure hereditary spastic paraplegia maps to chromosome 13q14. *Neurology.* 2002;59(12):1905-1909.

26. Zortea M, Vettori A, Trevisan C, et al. Genetic mapping of a susceptibility locus for disc herniation and spastic paraplegia on 6q23.3-q24.1. *J Med Genet.* 2002;39(6):387-390.

27. Inamori KI, Nakamura K, Shishido F, et al. Functional evaluation of novel variants of B4GALNT1 in a patient with hereditary spastic paraplegia and the general population. *Front Neurosci.* 2024;18:1437668.

28. Meijer I, Cossette P, Roussel J, et al. A novel locus for pure recessive hereditary spastic paraplegia maps to 10q22.1-10q24.1. *Ann Neurol.* 2004;56(4):579-582.

29. Miura S, Morikawa T, Fujioka R, et al. A novel frameshift mutation of DDHD1 in a Japanese patient with autosomal recessive spastic paraplegia. *Eur J Med Genet.* 2016;59(8):413-416.

30. Orlacchio A, Kawarai T, Gaudiello F, et al. New locus for hereditary spastic paraplegia maps to chromosome 1p31.1-1p21.1. *Ann Neurol.* 2005;58(3):423-429.

31. Boyle L, Rao L, Kaur S, et al. Genotype and defects in microtubule-based motility correlate with clinical severity in KIF1A-associated neurological disorder. *HGG Adv.* 2021;2(2).

32. Hata T, Nan H, Koh K, et al. A clinical and genetic study of SPG31 in Japan. *J Hum Genet.* 2022;67(7):421-425.

33. Stevanin G, Paternotte C, Coutinho P, et al. A new locus for autosomal recessive spastic paraplegia (SPG32) on chromosome 14q12-q21. *Neurology.* 2007;68(21):1837-1840.

34. Mannan A, Krawen P, Sauter S, et al. ZFYVE27 (SPG33), a novel spastin-binding protein, is mutated in hereditary spastic paraplegia. *Am J Hum Genet.* 2006;79(2):351-357.

35. Macedo-Souza L, Kok F, Santos S, et al. Reevaluation of a large family defines a new locus for X-linked recessive pure spastic paraplegia (SPG34) on chromosome Xq25. *Neurogenetics.* 2008;9(3):225-226.

36. Mari F, Berti B, Romano A, et al. Clinical and neuroimaging features of autosomal recessive spastic paraplegia 35 (SPG35): case reports, new mutations, and brief literature review. *Neurogenetics.* 2018;19(2):123-130.

37. Schule R, Bonin M, Durr A, et al. Autosomal dominant spastic paraplegia with peripheral neuropathy maps to chr12q23-24. *Neurology.* 2009;72(22):1893-1898.

38. Hanein S, Durr A, Ribai P, et al. A novel locus for autosomal dominant "uncomplicated" hereditary spastic paraplegia maps to chromosome 8p21.1-q13.3. *Hum Genet.* 2007;122(3-4):261-273.

39. Orlacchio A, Patrono C, Gaudiello F, et al. Silver syndrome variant of hereditary spastic paraplegia: A locus to 4p and allelism with SPG4. *Neurology.* 2008;70(21):1959-1966.

40. Synofzik M, Gonzalez M, Lourenco C, et al. PNPLA6 mutations cause Boucher-Neuhauser and Gordon Holmes syndromes as part of a broad neurodegenerative spectrum. *Brain.* 2014;137(Pt1):69-77.

41. Zhao G, Hu Z, Shen L, et al. A novel candidate locus on chromosome 11p14.1-p11.2 for autosomal dominant hereditary spastic paraplegia. *Chin Med J (Engl).* 2008;121(5):430-434.

42. Mao F, Li Z, Zhao B, et al. Identification and functional analysis of a SLC33A1: c.339T>G (p.Ser113Arg) variant in the original SPG42 family. *Hum Mutat.* 2015;36(2):240-249.

43. Landouré G, Zhu PP, Lourenço CM, et al. Hereditary spastic paraplegia type 43 (SPG43) is caused by mutation in C19orf12. *Hum Mutat.* 2013;34(10):1357-1360.

44. Orthmann-Murphy JL, Salsano E, Abrams CK, et al. Hereditary spastic paraplegia is a novel phenotype for GJA12/GJC2 mutations. *Brain.* 2009;132(Pt 2):426-438.

45. Elsaid MF, Ibrahim K, Chalhoub N, et al. NT5C2 novel splicing variant expands the phenotypic spectrum of Spastic Paraplegia (SPG45): case report of a new member of thin corpus callosum SPG-Subgroup. *BMC Med Genet.* 2017;18(1):33.

46. Boukhris A, Feki I, Elleuch N, et al. A new locus (SPG46) maps to 9p21.2-q21.12 in a Tunisian family with a complicated autosomal recessive hereditary spastic paraplegia with mental impairment and thin corpus callosum. *Neurogenetics.* 2010;11(4):441-448.

47. Tuysuz B, Bilguvar K, Kocer N, et al. Autosomal recessive spastic tetraplegia caused by AP4M1 and AP4B1 gene mutation: expansion of the facial and neuroimaging features. *Am J Med Genet A.* 2014;164A(7):1677-1685.

48. Pensato V, Castellotti B, Gellera C, et al. Overlapping phenotypes in complex spastic paraplegias SPG11, SPG15, SPG35 and SPG48. *Brain.* 2014;137(Pt7):1907-1920.

49. Neuser S, Brechmann B, Heimer G, et al. Clinical, neuroimaging, and molecular spectrum of TECPR2-associated hereditary sensory and autonomic neuropathy with intellectual disability. *Hum Mutat.* 2021;42(6):762-776.

50. Duerinckx S, Verhelst H, Perazzolo C, et al. Severe congenital microcephaly with AP4M1 mutation, a case report. *BMC Med Genet.* 2017;18(1):48.

51. Moreno-De-Luca A, Helmers SL, Mao H, et al. Adaptor protein complex-4 (AP-4) deficiency causes a novel autosomal recessive cerebral palsy syndrome with microcephaly and intellectual disability. *J Med Genet.* 2011;48(2):141-144.

52. Accogli A, Hamdan FF, Poulin C, et al. A novel homozygous AP4B1 mutation in two brothers with AP-4 deficiency syndrome and ocular anomalies. *Am J Med Genet A.* 2018;176(4):985-991.

53. Abou Jamra R, Philippe O, Raas-Rothschild A, et al. Adaptor protein complex 4 deficiency causes severe autosomal-recessive intellectual disability, progressive spastic paraplegia, shy character, and short stature. *Am J Hum Genet.* 2011;88(6):788-795.

54. Hardies K, May P, Djemie T, et al. Recessive loss-of-function mutations in AP4S1 cause mild fever-sensitive seizures, developmental delay and spastic paraplegia through loss of AP-4 complex assembly. *Hum Mol Genet.* 2015;24(8):2218-2227.

55. Zivony-Elboum Y, Westbroek W, Kfir N, et al. A founder mutation in Vps37A causes autosomal recessive complex hereditary spastic paraparesis. *J Med Genet.* 2012;49(7):462-472.

56. Nicita F, Stregapede F, Tessa A, et al. Defining the clinical-genetic and neuroradiological features in SPG54: description of eight additional cases and nine novel DDHD2 variants. *J Neurol.* 2019;266(11):2657-2664.

57. Spiegel R, Mandel H, Saada A, et al. Delineation of C12orf65-related phenotypes: a genotype-phenotype relationship. *Eur J Hum Genet.* 2014;22(8):1019-1025.

58. Tesson C, Nawara M, Salih MA, et al. Alteration of fatty-acid-metabolizing enzymes affects mitochondrial form and function in hereditary spastic paraplegia. *Am J Hum Genet.* 2012;91(6):1051-1064.

59. Elsayed LEO, Mohammed IN, Hamed AAA, et al. Hereditary spastic paraplegias: identification of a novel SPG57 variant affecting TFG oligomerization and description of HSP subtypes in Sudan. *Eur J Hum Genet.* 2016;25(1):100-110.

60. Dor T, Cinnamon Y, Raymond L, et al. KIF1C mutations in two families with hereditary spastic paraparesis and cerebellar dysfunction. *J Med Genet.* 2014;51(2):137-142.

61. Novarino G, Fenstermaker A, Zaki M, et al. Exome sequencing links corticospinal motor neuron disease to common neurodegenerative disorders. *Science.* 2014;343(6170):506-511.

62. Sakamoto M, Kurosawa K, Tanoue K, et al. A heterozygous germline deletion within USP8 causes severe neurodevelopmental delay with multiorgan abnormalities. *J Hum Genet.* 2024;69(2):85-90.

63. Chukhrova A, Akimova I, Shchagina O, et al. A new case of infantile-onset hereditary spastic paraplegia with complicated phenotype (SPG61) in a consanguineous Russian family. *Eur J Neurol.* 2019;26(5):e61-e62.

64. Cogan G, Zaki M, Issa M, et al. Biallelic variants in ERLIN1: a series of 13 individuals with spastic paraparesis. *Hum Genet.* 2024:Epub ahead of print.

65. Kortum F, Jamra RA, Alawi M, et al. Clinical and genetic spectrum of AMPD2-related pontocerebellar hypoplasia type 9. *Eur J Hum Genet.* 2018;26(5):695-708.

66. Ölmez A ÇG, Karaer K. Early onset disease, anarthria, areflexia, and dystonia can be the distinctive features of SPG64, a very rare form of hereditary spastic paraplegias. *Am J Med Genet A.* 2022;188(9):2712-2717.

67. Kettwig M, Elpeleg O, Wegener E, et al. Compound heterozygous variants in PGAP1 causing severe psychomotor retardation, brain atrophy, recurrent apneas and delayed myelination: a case report and literature review. *BMC Neurol.* 2016;16:74.

68. Williams C, Jiang YH, Shashi V, et al. Additional evidence that PGAP1 loss of function causes autosomal recessive global developmental delay and encephalopathy. *Clin Genet.* 2015;88(6):597-599.

69. Lacy S, Bonnemann C, Buzney E, et al. Identification of FLRT1, FLRT2, and FLRT3: a novel family of transmembrane leucine-rich repeat proteins. *Genomics.* 1999;62(3):417-426.

70. Sadr Z, Ghasemi A, Rohani M, et al. Three Iranian patients with rare subtypes of hereditary spastic paraplegia (HSP): SPG76, SPG56, and SPG69. *Neurogenetics.* 2024;26(1):12.

71. La Fay C, Hoebeke C, Juzaud M, et al. Deep phenotyping of MARS1 (interstitial lung and liver disease) and LARS1 (infantile liver failure syndrome 1) recessive multisystemic disease using Human Phenotype Ontology annotation: Overlap and differences. Case report and review of literature. *Eur J Med Genet.* 2021;64(11):104334.

72. Boutry M, Morais S, Stevanin G. Update on the Genetics of Spastic Paraplegias. *Curr Neurol Neurosci Rep.* 2019;19(4):18.

73. Esteves T, Durr A, Mundwiller E, et al. Loss of association of REEP2 with membranes leads to hereditary spastic paraplegia. *Am J Hum Genet.* 2014;94(2):268-277.

74. Rinaldi C, Schmidt T, Situ A, et al. Mutation in CPT1C Associated With Pure Autosomal Dominant Spastic Paraplegia. *JAMA Neurol.* 2015;72(5):561-570.

75. Lossos A, Stumpfig C, Stevanin G, et al. Fe/S protein assembly gene IBA57 mutation causes hereditary spastic paraplegia. *Neurology.* 2015;84(7):659-667.

76. Gan-Or Z, Bouslam N, Birouk N, et al. Mutations in CAPN1 Cause Autosomal-Recessive Hereditary Spastic Paraplegia. *Am J Hum Genet.* 2016;98(5):1038-1046.

77. Yang Y, Liu W, Fang Z, et al. A Newly Identified Missense Mutation in FARS2 Causes Autosomal-Recessive Spastic Paraplegia. *Hum Mutat.* 2016;37(2):165-169.

78. Estrada-Cuzcano A, Martin S, Chamova T, et al. Loss-of-function mutations in the ATP13A2/PARK9 gene cause complicated hereditary spastic paraplegia (SPG78). *Brain.* 2017;140(2):287-305.

79. Rydning SL, Backe PH, Sousa MML, et al. Novel UCHL1 mutations reveal new insights into ubiquitin processing. *Hum Mol Genet.* 2017;26(6):1031-1040.

80. Lin X, Su H, Dong E, et al. Stop-gain mutations in UBAP1 cause pure autosomal-dominant spastic paraplegia. *Brain.* 2018;142(8):2238-2252.

81. Ahmed MY, Al-Khayat A, Al-Murshedi F, et al. A mutation of EPT1 (SELENOI) underlies a new disorder of Kennedy pathway phospholipid biosynthesis. *Brain.* 2017;140(3):547-554.

82. Vaz F, McDermott J, Alders M, et al. Mutations in PCYT2 disrupt etherlipid biosynthesis and cause a complex hereditary spastic paraplegia. *Brain.* 2019;142(11):3382-3397.

83. Ghosh SG, Lee S, Fabunan R, et al. Biallelic variants in HPDL, encoding 4-hydroxyphenylpyruvate dioxygenase-like protein, lead to an infantile neurodegenerative condition. *Genet Med.* 2021;23(3):524-533.

84. Verdura E, Rodriguez-Palmero A, Velez-Santamaria V, et al. Biallelic PI4KA variants cause a novel neurodevelopmental syndrome with hypomyelinating leukodystrophy. *Brain.* 2021;144(9):2659-2669.

85. Kim Y, Kim S, Kim K, et al. Age-dependent gait abnormalities in mice lacking the Rnf170 gene linked to human autosomal-dominant sensory ataxia. *Hum Mol Genet.* 2015;24(25):7196-7206.

86. Lemire G, Ito YA, Marshall AE, et al. ABHD16A deficiency causes a complicated form of hereditary spastic paraplegia associated with intellectual disability and cerebral anomalies. *Am J Hum Genet.* 2021;108(10):2017-2023.

87. Tabara LC, Al-Salmi F, Maroofian R, et al. TMEM63C mutations cause mitochondrial morphology defects and underlie hereditary spastic paraplegia. *Brain.* 2022;145(9):3095-3107.

88. Schob C, Hempel M, Safka Brozkova D, et al. Dominant KPNA3 Mutations Cause Infantile-Onset Hereditary Spastic Paraplegia. *Ann Neurol.* 2021;90(5):738-750.

89. Deng R, Medico-Salsench E, Nikoncuk A, et al. AMFR dysfunction causes autosomal recessive spastic paraplegia in human that is amenable to statin treatment in a preclinical model. *Acta Neuropathol.* 2023;146(2):353-368.

90. Srivastava S, Shaked H, Gable K, et al. SPTSSA variants alter sphingolipid synthesis and cause a complex hereditary spastic paraplegia. *Brain.* 2023;146(4):1420-1435.
